# Vagal nerve stimulation for the management of long COVID symptoms

**DOI:** 10.1016/j.imj.2024.100149

**Published:** 2024-11-08

**Authors:** Malik W.Z. Khan, Muhammad Ahmad, Salma Qudrat, Fatma Afridi, Najia Ali Khan, Zain Afridi, Fahad, Touba Azeem, Jibran Ikram

**Affiliations:** a Yale University School of Medicine, New Haven, Connecticut 06520, USA; b Khyber Medical College, Peshawar 25120, Khyber Pakhtunkhwa, Pakistan; c Department of Cardiology, Cleveland Clinic Foundation, Ohio 44195, USA

**Keywords:** Long COVID, COVID-19, Invasive vagal nerve stimulation, Non-invasive vagal nerve stimulation, Transcutaneous vagal nerve stimulation

## Abstract

This review investigates the therapeutic potential of vagal nerve stimulation (VNS) in managing long COVID, a condition marked by persistent symptoms following acute SARS-CoV-2 infection. Long COVID manifests as ongoing fatigue, cognitive impairment, and autonomic dysfunction, hypothesized to arise from sustained inflammatory and neurological dysregulation. The vagus nerve, central to modulating systemic inflammation and autonomic homeostasis, represents a promising therapeutic target for symptom alleviation through VNS. A comprehensive literature search was conducted across PubMed, Scopus, and Web of Science to identify studies evaluating VNS in the context of long COVID. Preliminary evidence from small-scale pilot studies suggests VNS may attenuate systemic inflammation through activation of the cholinergic anti-inflammatory pathway (CAP), thus restoring autonomic balance and ameliorating symptoms such as fatigue, cognitive dysfunction, and anxiety. In targeting the inflammatory cascade that underlies both acute COVID-19 pathophysiology and its prolonged sequelae, VNS holds potential as an innovative intervention for persistent post-viral symptoms. While these initial findings indicate promise, current data remain limited in scope and robustness, underscoring the need for larger, controlled trials to validate the efficacy and mechanisms of VNS in long COVID management. Establishing a clearer understanding of VNS's impact on inflammation and autonomic regulation in this context is crucial to inform clinical guidelines and therapeutic strategies for long COVID, potentially offering a targeted approach for mitigating this disabling condition.

## Highlights

• Vagal nerve stimulation (VNS) has the potential to alleviate persistent long COVID symptoms.
• The sensory receptors on the vagus nerve can be activated by the cytokine storm associated with COVID-19, contributing to the “involuntary sickness response” that leads to chronic symptoms in long COVID.
• Non-invasive VNS techniques (nVNS) provide safe options for managing persistent COVID-19 symptoms with minimal side effects.
• The current evidence for VNS as a potential treatment for long COVID is limited and largely based on small pilot studies, underscoring the need for future research to evaluate its effectiveness.

## Graphical abstract

**Figure:**
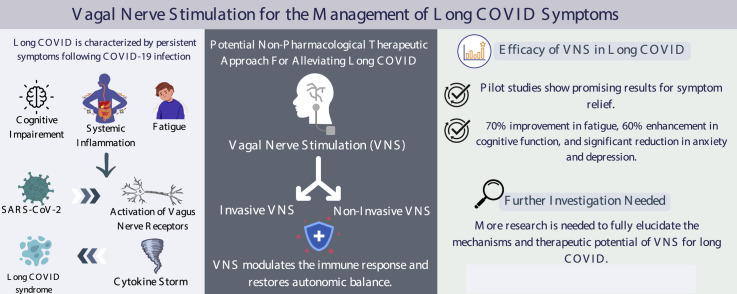

## 1 Introduction

The novel severe acute respiratory syndrome coronavirus 2 (SARS-CoV-2) is responsible for causing coronavirus disease 2019 (COVID-19), a disease characterized by excessive inflammation in multiple organs, causing a broad spectrum of manifestations [1]. While most COVID-19 symptoms improve over time, some patients experience persistent symptoms including fatigue, dyspnea, anxiety, depression, and chest pain for months, extending to over 6 months in some individuals. This condition is described as long COVID, previously known as post-acute sequelae of COVID-19 or post-COVID-19 syndrome [2,3]. According to estimates as of June 2022, the prevalence of long COVID is between 10% – 35% in the population who had COVID and it is up to 85% for hospitalized patients [3]. The risk factors for long COVID include hospitalizations especially in the Intensive Care Unit (ICU) and increasing age with pre-existing medical conditions like pneumonia and venous thromboembolism. Younger and healthier people are more likely to recover faster from long COVID [3]. Women are more susceptible to long COVID syndrome due to their increased risk of autoimmune disorders and stronger immune responses [3]. This complex condition affects various systems and can range from mild to severe symptoms. Common residual effects of COVID-19 include fatigue, difficulty breathing, chest pain, cognitive issues, joint pain, and a decline in quality of life [4]. Studies suggest that the immune response to SARS-CoV-2 infection, leading to cytokine production and a procoagulant state, may contribute to these long-term symptoms [5,6]. Similar persistent symptoms have been observed in survivors of previous coronavirus outbreaks, such as the SARS epidemic in 2003 and the Middle East Respiratory Syndrome (MERS) outbreak in 2012, further highlighting the concern for the long-term effects of COVID-19 [7]. This review aims to summarize the current evidence on the potential role of vagal nerve stimulation (VNS) in the management of long COVID symptoms, providing a comprehensive overview of the underlying mechanisms, clinical studies, and future research directions in this emerging field.

## 2 Pathophysiology of long COVID: the cytokine storm

**Fig. 1:**
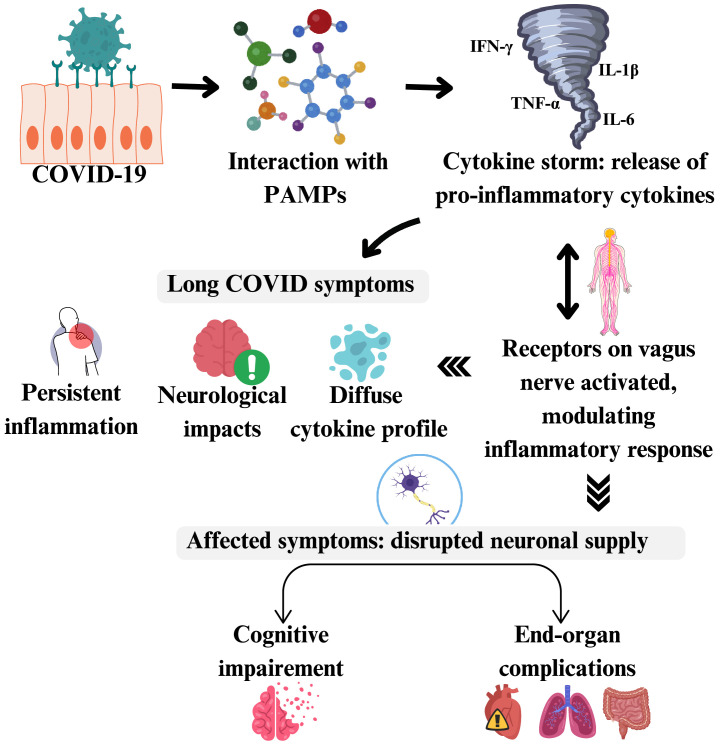
A concise summary illustrating the pathophysiology of COVID-19 and its impacts.

As the management of COVID-19 progressed, it became evident that the disease's pathological effects are largely attributed to an abrupt and intense inflammatory response, often referred to as the cytokine storm [8]. The pathophysiology of cytokine storms in COVID-19 shares similarities with the mechanism of sepsis [9]. Both the conditions result in the hype of inflammatory cytokines including interleukin-6 (IL-6) and tumor necrosis factor-α (TNF-α) [10] thrombocytopenia, vascular microthrombosis and multi-organ dysfunction [9]. In sepsis, the interaction between pathogen-associated molecular patterns (PAMPs) and their receptors on immune cells triggers the release of cytokines. Excessive immune response to an overwhelming amount of harmful antigens results in a "storm" of cytokines, causing excessive inflammation and potential damage to organs. This same mechanism is involved in the cytokine release syndrome and the inflammatory reflex in COVID-19, which is controlled by the vagus nerve, and plays a crucial role in the body's defense against bacterial and viral infections [11,12]. Numerous investigations have verified that people with long COVID display an unusual, diffuse inflammatory cytokine profile that lasts for months and is not present in COVID-19 survivors who are asymptomatic [13]. Fig. 1 illustrates the pathophysiological mechanisms of COVID-19 and their implications for long-term consequences.

According to Xie et al. [14], individuals experiencing persistent COVID-19 symptoms face a heightened risk of various cardiovascular conditions such as cerebrovascular disorders, dysrhythmias, ischemic and non-ischemic heart disease, pericarditis, myocarditis, heart failure, and thromboembolic disease. In a separate study, Xie et al. [15] discovered that individuals in the post-acute phase of the disease have an increased risk (HR 1.40, 95% CI 1.36–1.44) and an excessive burden of diabetes mellitus, as well as an excess burden of incident antihyperglycemic use, compared to a contemporary control group. It has been suggested that long COVID may encompass various syndromes, making it challenging to predict the extent of recovery [16]. Based on available information, there is a suggestion that individuals with long COVID may experience ongoing issues in the brainstem, leading to vagus nerve signaling dysfunction [17,18]. This dysfunction of the vagus nerve or brainstem is considered a potential neurological factor contributing to cognitive impairments associated with long COVID. Some of the most common symptoms include memory impairment, executive dysfunction, and language dysfunction [19]. Supporting this hypothesis, impaired vagal activity has been observed in individuals suffering from long COVID-19 [20], further highlighting the malfunctioning signaling of the vagus nerve or brainstem [21,22]. A study of approximately 1,000 patients with SARS-CoV-2 infection found that 26% experienced mild cognitive impairment after recovering from the initial illness [23]. Research indicates that individuals with a history of COVID-19 infection often struggle with attention, multitasking, and exhibit reduced mental efficiency [19]. Furthermore, a large cohort study involving over 200,000 patients revealed that the risk of developing dementia within 6 months after COVID-19 infection was more than twice as high compared to those who had influenza during the same period [24]. A study published in *Lancet* compared 270 patients with long-COVID to those without and uninfected controls, and found significant cognitive slowing in the patients with long-COVID [25]. The study also revealed that the cognitive slowing associated with long-COVID did not resolve over time [25].

Additionally, recent meta-analytical research has shown that transcutaneous auricular vagus nerve stimulation (taVNS) can effectively alleviate symptoms in individuals with depression [26]. Studies have also indicated that taVNS has the potential to enhance attention, memory, decision-making, and cognitive control [27]. These findings suggest that VNS to mitigate inflammation may have beneficial implications in addressing the clinical requirements of hospitalized COVID-19 patients [28]. Importantly, these cognitive processes are also impacted by long COVID [29].

While various pharmacological therapies, including corticosteroids, IL-6 inhibitors like tocilizumab, PD-1/PD-L1 checkpoint inhibitors, and intravenous immunoglobulins, have been used to control the cytokine storm in COVID-19, nonpharmacological approaches have not been extensively studied. The use of VNS to modulate cholinergic anti-inflammatory pathways (CAPs) in the treatment of inflammatory diseases and sepsis suggests that VNS could be a promising therapeutic option for managing the inflammatory processes associated with COVID-19 infection. Human studies have demonstrated that VNS can effectively decrease or halt the elevation of proinflammatory cytokines in the bloodstream, including TNF-α, IL-1β, IL-6, and interferon-γ. It is important to note that the specific cytokines affected may vary depending on the specific disease conditions being investigated [[30], [31], [32]].

## 3 Long COVID and the vagus nerve

COVID-19 can affect the normal body state when it gains entry through the viral pathway, which leads to a cytokine storm [8]. The SARS-CoV-2 primarily enters the body through the olfactory or gustatory pathway and targets receptors on cranial nerves IX and X, particularly affecting myelin-forming nerve cells and oligodendrocytes, leading to impaired vital functions. It binds to various receptors located in the neurons (brain stem) such as angiotensin-converting enzyme 2 (ACE2), neuropilin 1 (NRP1), and transmembrane protease serine 2 (TMPRSS2). ACE2 receptors are widely present on the neuronal sheath, which carries sensory information from the respiratory and gustatory sections [33]. When the virus invades the nerve, it disrupts the transmission of signals to the brain, resulting in a decline in the parasympathetic activity of the vagus nerve. This leads to respiratory distress, coagulation of small pulmonary vessels, and subsequent inflammation. Additionally, the invasion of the vagus nerve (a parasympathetic nerve) by SARS-CoV-2 creates an imbalance between the sympathetic and parasympathetic systems, leading to activation of the sympathetic system and systemic inflammation through a cluster of differentiation (CD4) [2]. Since the body is composed of chemicals and electricity, this dysautonomia disrupts the balance between chemicals and ions, leading to the release of cytokines (IL-6, TNF, IL-11, etc.), which disturbs the body's normal homeostatic state [1]. This is also evident from the study that sensory receptors on the vagus nerve can be activated by the cytokine storm and further appreciate the “involuntary sickness response” that if continued, results in chronic symptoms as in long COVID [17]. Therefore, restoring the normal homeostatic balance is crucial for relieving the symptoms caused by the cytokine storm.

The other pathway that the virus takes on is the blood-brain barrier and its transfer across the glossopharyngeal nerve which lies near the branches of the vagus nerve. This close relation affects the efficiency of vagus nerve signaling and contributes to the neurological factor contributing to the development of long COVID symptoms [34]. Thus, there are a lot of similarities between vagus nerve functioning and the incidence of long COVID symptoms [35]. Neuronal efferent supply to the heart, lungs, gastrointestinal tract, and neurological domains (sleep, nausea, vomiting, breathing) by the vagus and afferent supply to the brain from the brainstem is disrupted by long COVID, leading to affective symptoms and cognitive defects [36].

## 4 Technological advances in precision medicine: implications for vagal nerve stimulation in long COVID

The technological advancement in medicine is fascinating. In the realm of precision medicine, fiber optic-based biosensors represent a technological advancement that enhances diagnostic accuracy and targeted therapy. These biosensors, with their ability to detect disease biomarkers and deliver precise treatments such as photothermal therapy, exemplify the integration of personalized medical technologies. One notable use of fiber optic-based biosensors is in oncology, to precisely detect cancer cells through the principle of antigen-antibody binding, that can be seen on the surface of fiber optic. Some biomarkers have a photothermal effect feature, causing localized temperature elevation up to 41.4 degree centigrade. This temperature causes breakdown of phospholipid bilayers that induce cancer cells apoptosis [37]. Another kind of biosensor uses a refractive index to detect SARS-CoV-2 in patients. A change in SARS-CoV-2 concentration can cause alteration in the refractive index, which is detected by biosensors. However, this method has a minimal limit for detection [38].

Similarly, VNS can be viewed as a precision intervention for long COVID symptoms, tailored to modulate specific physiological responses, aligning with the personalized approach in managing post-COVID conditions. The pathophysiology underlying long COVID symptoms is not well-known, however, the associated symptoms lead to a substantial decline in quality of life. By modulating parasympathetic activity and thus influencing inflammatory pathways, VNS serves as a versatile treatment modality for a diverse array of medical and neurological conditions. The potential efficacy of VNS in restoring autonomic balance disrupted by COVID-19 infection has paved the way for non-pharmacological therapeutic interventions in the management of symptoms associated with long COVID. Bailey and Bremer were the pioneering researchers who demonstrated significant changes in electroencephalography due to VNS [39]. The potential of VNS to manage both well-established medical conditions like depression, anxiety, and migraine, and emerging challenges like long COVID is remarkable. There are 2 types of VNS: invasive vagal nerve stimulation (iVNS) and non-invasive vagal nerve stimulation (nVNS), which further has 2 types: (1) taVNS and (2) transcutaneous cervical VNS (tcVNS).

VNS has been a healthcare practice for over 30 years and has been used to treat various conditions such as migraines, Alzheimer's disease, schizophrenia, tinnitus, anxiety, and cognitive disorders. Early research by Adam Broncel showed that manual suppression of the vagus nerve provided relief from seizure attacks in epileptic patients [39]. Studies have shown that over 50% of patients experience partial to complete recovery from seizures with VNS [40]. iVNS therapy received FDA approval in 1990 for the treatment of epilepsy and depression [39]. Following FDA approval for epilepsy, iVNS has also been authorized for the treatment of depression and obesity [40]. Notably, iVNS has shown significant improvements in depression, anxiety, and mood assessment scores, with an average increase of 35% in depression scores, 35% in anxiety scores, and 25% in mood assessment scores [40]. VNS is approved for all ages and genders and can treat different types of seizures, including focal, localized, partial, and complex seizures [40]. Despite the outstanding role of iVNS therapy in treating a variety of medical and neurological conditions, this therapy comes with multiple surgical and post-surgical complications and is extremely costly with the total costs of the surgery being around AU$500,000 [41].

The most frequently used stimulation devices today are the gammacore, electrocore, and the NEMOS cerbomed. The NEMOS cerbomed device is used more often at the site of the ear where it is used for the stimulation of the auricular branch of the vagus nerve, while the gammacore and the electrocore device is frequently used at the neck site where it is used for the stimulation of the cervical branch of vagus nerve [41]. Anatomically, the cutaneous afferent fibers of the vagus nerve are located on the tragus, concha, and cymba concha of the ear, and stimulation of these afferent fibers by the nVNS devices produces effects, including anti-inflammatory effects and decreased pain sensations, like the conventional invasive devices. Similarly, non-invasive stimulation of the cervical branch of the vagus nerve in the neck has also shown promising results, including antidepressant and anti-seizure effects [41]. The nVNS has also been implicated to be beneficial in the treatment of primary headache disorders. tcVNS provides a nonpharmacological, low-risk preventive measure in patients with paroxysmal hemicrania, hemicrania continua, cough headache, or short-lasting neuralgiform headache attacks (SUNCT/SUNA), who are unable to tolerate preventive medication [42]. Additionally, in a recent issue of Bioelectronic Medicine, it was reported that in pediatric patients with mild to moderate IBD, taVNS effectively reduced signs and symptoms without any notable side effects, thus potentially paving the way for new treatment options for both children and adults with IBD [4,43]. VNS has shown promising results in oligodendrocyte differentiation and myelin formation in rats, a possible way of treating multiple sclerosis. However further studies are necessary to validate the finding [44]. A review study states the anti-inflammatory role of VNS in reducing inflammatory biomarkers and the size of the infarct, thereby reducing the ventricular arrhythmias [45]. According to a study by Linnhoff et al. [46] non-invasive brain stimulation techniques (NIBS) like vagal nerve still and transcranial magnetic stimulation (TMS) show great success in improving cognitive function, reducing fatigue and reducing anxiety hence leading to improved quality of life. The factors that give nVNS an advantage over iVNS is the fact that nVNS is less costly and does not require surgical procedures for installation.

## 5 Role of vagus nerve stimulation in alleviating long COVID symptoms

Significant advancements have been reported in finding methods to treat acute COVID infection, yet there are very few or no fruitful therapies for lowering the severity of long COVID symptoms. Although the pathophysiology of long COVID symptoms is not yet very well explained, symptoms like fatigue, respiratory difficulty, and musculoskeletal pain are speculated to be a result of the inflammatory response and delayed immune response after the entry of SARS-CoV-2 into the vagus nerve [47]. VNS has shown positive outcomes to alleviate the symptoms of the long COVID through different mechanisms of action, including the inhibition of airway constriction via parasympathetic-sympathetic reflex arc, and the activation of CAPs to regulate the immune response to COVID-19 [48,49]. This is consistent with the findings from a recent prospective pilot study, whereby significant improvements in cognitive function, anxiety and depression, fatigue, and sleep were reported in female patients with long COVID, 10 days after starting treatment with tcVNS [50]. Other possible mechanisms explaining the effectiveness of VNS in treating long COVID symptoms include reduction of systemic inflammation, cerebral blood flow enhancement, neurotransmitter modulation, and restoring normal autonomic function [50]. Moreover, the HPA axis, which is responsible for the body's reaction to stress and regulates mood and vitality, is also affected by VNS, further implicating the use of vagal neuromodulation in mitigating inflammatory states associated with anxiety and depression [50]. Lorenza S. Colzato et al. [34] stated that tcVNS can invigorate the inert brainstem nuclei in patients suffering from long COVID and can improve the symptoms.

In COVID-19 patients, elevated levels of inflammatory biomarkers, including procalcitonin and D-dimer, have been associated with poor clinical outcomes and prognoses, such as respiratory collapse and mortality [29]. A prospective randomized control study conducted at Hospital Clinico Universitario Spain, by Tornero et al. [29] concluded that nVNS therapy shows positive results in lowering the levels of inflammatory biomarkers, particularly procalcitonin and C-reactive protein, and is feasible to be implemented in the hospital setting. Also, an open-label pilot study by Benjamin et al. involving 14 patients suffering from chronic fatigue syndrome related to long COVID has shown improved symptoms in the 8 patients, however, this concluded with the dire for further research to refine the conclusive validity of tcVNS [51]. Hence, the potential use of VNS to mitigate the inflammatory processes associated with long COVID, and to restore autonomic balance offers promise as an effective treatment for managing symptoms associated with long COVID, including fatigue, cardiovascular problems, and neurological conditions [36]. However, the underlying studies manifesting VNS for alleviating long COVID symptoms are still sparse and confined.

## 6 Safety and tolerance of VNS

VNS is generally regarded as safe and well-tolerated when used to treat chronic long COVID symptoms. Transcutaneous vagus nerve stimulation, including taVNS, has minimal side effects, such as local skin irritation (18.2%), headache (3.6%), and nasopharyngitis (1.7%), with a low dropout rate of 2.6% due to adverse events, according to a thorough systematic review involving 51 studies and 1,322 human subjects [52]. Additionally, dizziness, hoarseness of voice, and nasopharyngitis have been reported with non-invasive VNS.

The safety and viability of taVNS as a clinical intervention were reaffirmed by a comprehensive meta-analysis that included 177 trials and 6,322 participants. It also revealed no discernible variations in the incidence of adverse events between active taVNS and controls [53]. While surgically implanted VNS devices present risks like pain, infection, and transient issues with voice or swallowing, these are generally well-tolerated and diminish over time [54]. Overall, tVNS and iVNS techniques are encouraging options for treating protracted COVID-19 pilot trials or extrapolations from other illnesses, more research is necessary to clarify the precise symptoms; nevertheless, as most of the evidence on taVNS for long COVID comes from small processes by which it may improve symptoms, and to elucidate the safety and acceptability of the intervention in the population. There is ample evidence from studies indicating that VNS can effectively alleviate a variety of medical and neurological conditions including long COVID symptoms (Table 1).

**Table 1:** Summary of Studies on VNS Related to long COVID.

Study	Study Population	Study Design	Type of VNS	Outcome
Tornero et al. [29]	COVID-19 Positive	RCT	Non-invasive VNS	Lowered levels of inflammatory biomarkers like procalcitonin and CRP in COVID-19 patients
Colzato et al. [34]	Long COVID patients	Review Article	Transcutaneous auricular VNS (taVNS)	Transcutaneous auricular VNS (taVNS) is considered effective in boosting memory, attention, and vagal signaling
Toffa et al. [40]	Patients with Epilepsy	Critical Review	Implanted VNS	Over 50% of patients experienced partial to complete recovery from seizures
Toffa et al. [40]	Patients with Epilepsy	Critical Review	Implanted VNS	35% increase in depression scores, 35% in anxiety scores, 25% in mood assessment scores
Badran et al. [47]	COVID-19 positive	Pilot RCT	Transcutaneous auricular VNS (taVNS)	In one month,12 Long COVID patients self-administered taVNS at home safely
Zheng et al. [50]	Long COVID female patients	Pilot Study	Transcutaneous VNS	Significant improvements in cognitive function, anxiety, depression, fatigue, and sleep in female patients with long COVID
Natelson et al. [51]	Long COVID patients with chronic symptoms	Pilot Study	Transcutaneous VNS	Out of 14, only 6 couldn't achieve the criteria for primary outcomes—however, 8 remaining achieved.
Redgrave et al. [52]	Patients with epilepsy, migraine, tinnitus and depression	Systematic review	Transcutaneous VNS	18.2% local skin irritation, 3.6% headache, 1.7% nasopharyngitis, 2.6% dropout rate due to adverse events
Kim et al. [53]	Patients receiving taVNS	Systematic review and Meta-analysis	Transcutaneous auricular VNS (taVNS)	Adverse event rates between active taVNS and controls did not significantly differ
Révész et al. [54]	Patients suffering from refractory epilepsy	Prospective Cohort	Implanted VNS	Pain, infection, and transient voice/swallowing issues, generally well-tolerated and diminished over time

Abbreviations: nVNS, non-invasive vagus nerve stimulation; iVNS, invasive vagus nerve stimulation; tcVNS, transcutaneous vagus nerve stimulation; taVNS, transcutaneous auricular vagus nerve stimulation.

## 7 Conclusion

Vagus nerve dysfunction is considered a potential neurological factor contributing to cognitive impairments associated with long COVID. The invasion of the vagus nerve by COVID-19 infection causes an imbalance between the sympathetic and parasympathetic systems, leading to the activation of the sympathetic system and causing systemic inflammation. Boosting up the function of the vagus nerve can thus be one way to overcome this imbalance and repose the normal body state. Very few studies have confirmed this pragmatic achievement of VNS to relieve long COVID symptoms. Future investigations and clinical trials, involving large sample sizes of patients affected by long COVID, should be carried out to help pave the way for further advancement in therapeutic options for patients with long COVID.

## Funding

This research did not receive any specific grant from funding agencies in the public, commercial, or not-for-profit sectors.

## CRediT authorship contribution statement

**Malik W.Z. Khan:** Writing – review & editing, Writing – original draft, Validation, Supervision, Resources, Project administration, Methodology, Investigation, Formal analysis, Data curation, Conceptualization. **Muhammad Ahmad:** Writing – review & editing, Writing – original draft, Visualization, Validation, Software, Project administration, Methodology, Investigation, Formal analysis, Data curation. **Salma Qudrat:** Writing – review & editing, Writing – original draft, Visualization, Validation, Supervision, Resources, Project administration, Methodology, Investigation. **Fatma Afridi:** Writing – review & editing, Writing – original draft, Visualization, Validation, Resources, Methodology, Investigation, Conceptualization. **Najia Ali Khan:** Writing – review & editing, Writing – original draft, Visualization, Validation, Software, Resources, Methodology, Investigation, Conceptualization. **Zain Afridi:** Writing – review & editing, Writing – original draft, Visualization, Validation, Resources, Project administration, Methodology, Formal analysis. **Fahad:** Writing – review & editing, Writing – original draft, Visualization, Validation, Software, Resources, Project administration, Methodology, Conceptualization. **Touba Azeem:** Writing – review & editing, Writing – original draft, Visualization, Validation, Software, Resources, Project administration, Investigation. **Jibran Ikram:** Writing – review & editing, Writing – original draft, Visualization, Validation, Software, Resources, Investigation.
